# A Literature Review on Thyrotoxic Periodic Paralysis

**DOI:** 10.7759/cureus.10108

**Published:** 2020-08-29

**Authors:** Qasim Z Iqbal, Muhammad Niazi, Zeeshan Zia, Saud Bin Abdul Sattar

**Affiliations:** 1 Internal Medicine, Northwell Health, New York, USA

**Keywords:** thyrotoxicity, endocrinology, hyperthyroidism, reversible cause of muscle weakness

## Abstract

Thyrotoxic periodic paralysis (TPP) is a rare disease of the muscles that presents with painless weakness of the muscles. The patients usually have hypokalemia and hyperthyroidism with elevations in the level of triiodothyronine (T3) and thyroxine (T4). The muscle weakness is usually transient, and the patients in many cases suffer from recurrent episodes of muscle paralysis. This flaccid muscle paralysis predominantly affects the proximal and lower extremities group of muscles more than the distal and upper extremity muscles. This condition is one of the drastic complications of Graves's disease and, unfortunately, may require admission and treatment in the critical care units. It is often not recognized during the initial attack in the American population as the prevalence is very low among the Caucasian population and people from North America. However, while the prevalence is extremely low in the Caucasian population, it is known to be 10 times more common among the Asian population when compared with the Caucasian population. Furthermore, while the diseases of the thyroid gland are more common in females, this rare disease predominantly affects male sex. It is treated by reversing the hypokalemia, which can in itself prove to be fatal if not corrected quickly, and this is followed by treatment to restore the euthyroid state. A literature review on this reversible cause of muscle weakness is very important to better understand this disease.

## Introduction and background

Thyrotoxic periodic paralysis (TPP) is a rare disease of the muscles. It presents with acute painless weakness of the muscles. The muscle weakness is recurrent and reversible, lasting from hours to sometimes days. This muscle weakness is known to be more severe in the proximal and lower extremity muscles. It is usually not recognized during the initial attack as the prevalence is very low among various populations especially the Caucasian population. Most cases of TPP are seen in patients with Grave's disease. Interestingly, despite the general higher incidence of hyperthyroidism found in females, this rare disease is more common in males. The patients would have a combination of hypokalemia and hyperthyroidism. The management focuses on the earlier and optimal replacement of potassium to avoid fatal complications of hypokalemia such as cardiac arrhythmias. This is then followed by restoration of the euthyroid state in the patient. Our review emphasizes the importance of both diagnosing and treating TPP early as this would decrease both mortality and morbidity in these patients.

## Review

TPP is a rare disease of the muscles that presents with an acute painless weakness of the muscles. The patients affected usually have both hypokalemia and hyperthyroidism. This muscle weakness is found to be more severe in the proximal muscles and in the lower extremities. TPP is often not recognized at first attack due to a very low prevalence among the Caucasian population and since it is usually associated with mild symptoms of hyperthyroidism [1].

This disease is found to be more common among the Asian population. It occurs in 0.1-0.2% of hyperthyroid patients in North America and is 10 times more common among the Asian population and males [2]. The high incidence of TPP among the Asian population is associated with the human leukocyte antigen DRw8 isotype (HLA-DRw8). This shows that the main cause may be genetic, but the exact mechanism behind is still unknown [3].

The control of potassium ion in the body is mainly by two ion channels, namely sodium-potassium (Na-K) pump and inward rectifying potassium channel (Kir). Both these channels work together in tight control to maintain serum potassium levels. Na-K pump pumps sodium into the cells (influx), whereas Kir channels control outward flow of potassium ions, i.e., efflux from the cells [4]. The latter channels are inhibited by catecholamines and insulin, causing hypokalemia. Mutations of Kir can also contribute to hypokalemia. According to one recent study, loss of function mutation of the Kir channel (Kir 2.6) was found in approximately 33% of patients with TPP [5]. The severity of the disease is not found to be correlated with the TPP, although muscle paralysis is known to be resolved once the euthyroid state is achieved. Hyperthyroid patients are known to have a high beta-adrenergic activity that pumps potassium inside the cells, causing hypokalemia that results in muscle weakness. Total potassium levels inside the body remain the same. There is no excessive gastrointestinal (GI) or urinary loss of potassium [2]. The best diagnostic test to diagnose the cause behind hypokalemia is 24-hour urine potassium and potassium-to-creatinine ratio (K/Cr). Former is actually impractical in acute settings where potassium is actively being replete in the patients, whereas K/Cr is very useful, and a value of less than 13 in the setting of hypokalemia favors either GI loss or transcellular shift [6,7].

Other precipitants of TPP include hyperinsulinemia state such as carbohydrate-rich meals, obesity, and catecholamine surge (recurrent episodes are more common during the early morning) [2,3]. There is a reported case where a person had muscle paralysis every Monday possibly because of the intake of large amounts of meals on weekends [8]. Hypokalemia can also be caused by androgens as they increase the activity of Na-K pump, whereas the estrogen and progesterone decrease it [9,10]. This can be one of the reasons behind TPP being common among males. Hypomagnesemia and hypokalemia are also found to be associated with TPP. Similarly, there are case reports where patients developed TPP after high-dose steroids [11]. In another instance, patients developed TPP after starting antiretroviral therapy for AIDS [12] or interferon-gamma therapy [13].

The diagnosis is based on clinical presentation and by excluding other disorders associated with low potassium. High serum triiodothyronine (T3) and thyroxine (T4), low serum thyroid-stimulating hormone (TSH) levels, and thyroid uptake scan showing symmetric diffuse uptake are all part of the diagnostic evaluation. Furthermore, abnormal electrocardiogram (ECG) and electromyogram (EMG) findings accompanied by biochemical evidence of hypokalemia can further help in diagnosis. Electromyography study of the muscles usually shows a myopathic pattern of the muscle weakness that completely resolves during remission. There are reported cases where TPP results in rhabdomyolysis with normal creatinine phosphokinase levels. Muscle biopsy of skeletal muscles, if performed, has shown different structural changes, including, but are not restricted to, sarcolemmal nuclear proliferation, vacuolation, atrophy of muscle fibers, fatty infiltration, and mitochondrial changes on the histology [14,15]. All these structural changes interfere with muscle contraction, leading to its paralysis.

One of the important and most feared complications associated with thyrotoxicosis that interestingly might be overlooked frequently is hypokalemia-induced cardiac arrhythmias. However, these are found to be more common among patients with pre-existing cardiac diseases. According to one study by Manthri et al. [16], in which they studied the incidence and prevention of TPP in patients of hyperthyroidism, 44 of 100 patients presented with muscle weakness and one of them died due to ventricular arrhythmia with ECG findings consistent with hypokalemia.

The disease severity can vary from mild weakness to quadriplegia to total paralysis. There is in most cases no family history of muscle paralysis. Interestingly, the bulbar, respiratory, and ocular muscles are in most cases spared. There is, however, an interesting case report of a patient who exhibited upper motor neuron signs (hyperreflexia, ankle clonus, positive Babinski sign), which resolved after treatment of the underlying hyperthyroidism [17,18]. The deep tendon reflexes in these patients can be diminished to absent. There are some case reports where patients would present acutely with monoplegia highly mimicking a stroke [19]. Cognitive and sensory functions usually remain normal. The start of muscle paralysis frequently coincides with hyperthyroidism, but there are reported cases where TPP is seen in patients with subclinical hyperthyroidism, which was later diagnosed when further workup for hyperthyroidism is done.

The differential diagnosis of TPP would include familial hypokalemic periodic paralysis (FHPP). Since the patient needs to be hyperthyroid for TPP, it occurs at a later age when compared to FHPP. In a study, 605 of the patient developed paralysis before the age of 16 years in FHPP and 79% of patients developed paralysis in-between the ages of 29-39 years. Importantly, normal serum potassium levels in between episodes of muscle weakness differentiate TPP with FHPP. Moreover, FHPP is distinguished by the presence of ionic channel genes such as calcium voltage-gated channel subunit alpha 1 S (CACNA1S), sodium voltage-gated channel alpha subunit 4 (SCN4A), and potassium voltage-gated channel subfamily E regulatory subunit 3 (KCNE3) [20].

The management of TPP can be divided into acute and definite treatment. While managing the acute episodes, potassium can be repleted intravenously to relieve the symptoms of hypokalemia. However, unfortunately, this can result in rebound hyperkalemia when potassium shifts extracellularly [20,21]. This is because patients with TPP do not actually have a total body deficiency of potassium; hence, close attention must be given to potassium replacement, as overly aggressive treatment can result in rebound hyperkalemia. Therefore, for this reason, no more than 60 milliequivalents of potassium should be given in the first 24 hours. Non-selective beta-blockers such as propranolol can also be used to block the effect of catecholamines on ion channels. The definitive therapy includes surgery (thyroidectomy), radioactive ablation, or anti-thyroid medications such as methimazole or propylthiouracil. Lastly, currently, there is no evidence of the benefit of using potassium supplements prophylactically to avoid TPP episodes.

## Conclusions

TPP is a rare disease of muscles that presents with acute painless weakness of muscles predominantly affecting the proximal and lower extremity muscles. It is usually found in patients with hypokalemia and hyperthyroidism. This condition is very rare in Caucasian populations and is very commonly misdiagnosed in western countries because of its similarities to familial periodic paralysis. It can be an initial presentation of thyrotoxicosis and the muscle weakness is reversible; hence, a better understanding of the disease would help us in both diagnosing and treating it early. In acute episodes, potassium can be given carefully through intravenous access, and non-selective beta-blockers can be used to block the effect of catecholamines on ion channels, resulting in the resolution of muscle weakness. The definitive therapy, however, could include surgery (thyroidectomy), radioactive ablation, or anti-thyroid medications such as methimazole or propylthiouracil.
